# Alterations in Gut Microbiota as Early Biomarkers for Predicting Inflammatory Bowel Disease Onset and Progression: A Systematic Review

**DOI:** 10.7759/cureus.58080

**Published:** 2024-04-11

**Authors:** Kusalik Boppana, Naiela E Almansouri, Saloni Bakkannavar, Youmna Faheem, Amisha Jaiswal, Kainaat Shergill, Tuheen Sankar Nath

**Affiliations:** 1 Internal Medicine, California Institute of Behavioral Neurosciences & Psychology, Fairfield, USA; 2 Internal Medicine, Manipal University, Manipal, IND; 3 Internal Medicine, University of Tripoli, Tripoli, LBY; 4 Pediatrics, California Institute of Behavioral Neurosciences & Psychology, Fairfield, USA; 5 Medicine, New Medical Center Royal Hospital, Abu Dhabi, ARE; 6 Surgery, California Institute of Behavioral Neurosciences & Psychology, Fairfield, USA; 7 Medicine, Maharishi Markandeshwar Institute of Medical Sciences and Research, Mullana, IND; 8 Surgical Oncology, California Institute of Behavioral Neurosciences & Psychology, Fairfield, USA

**Keywords:** inflammatory bowel disease diagnosis, gut microbiome, biomarkers, inflammatory bowel disease, ulcerative colitis, crohn`s disease

## Abstract

Inflammatory bowel disease (IBD) is a chronic ailment impacting the digestive system, triggered by an unusual reaction of the immune system. It includes two types of diseases: ulcerative colitis and Crohn’s disease. Nonetheless, the diagnosis and evaluation of disease progression in IBD are difficult due to the absence of distinct indicators. While conventional biomarkers from blood plasma and feces, such as C-reactive protein, fecal calprotectin, and S100A12, can be employed to gauge inflammation, they are not exclusive to IBD. There is a broad consensus that intestinal microorganisms significantly contribute to the onset of intestinal imbalance, a condition intimately linked with the cause and development of IBD. Numerous studies have indicated that the makeup of intestinal microorganisms varies between individuals with IBD and those who are healthy, particularly concerning the diversity of microbes and the proportional prevalence of certain bacteria. A total of 1475 records underwent examination. Following the eligibility assessment, 17 reports were considered. The final review encompassed 12 studies, as five articles were excluded due to insufficient details regarding cases, controls, and comparability. This article suggests that gut microbiota has potential biomarkers for the noninvasive evaluation of IBD activity. Recognizing the microbiome linked with disease activity paves the way for the development of a group of microbiota-derived indicators to evaluate the initiation and advancement of IBD. This article discusses whether changes in gut microbial composition can serve as early indicators of IBD onset and progression.

## Introduction and background

In the United States, approximately one million people are affected by inflammatory bowel disease (IBD), with roughly 30,000 new cases being identified annually. The occurrence of both ulcerative colitis (UC) and Crohn's disease (CD) is evenly distributed among these cases [1].

IBDs are persistent inflammation-related conditions that primarily manifest in two forms: UC and CD. The global prevalence of IBD has been consistently on the rise, largely in line with industrial progression, leading to an increase in healthcare costs [2]. The prevalence of IBD differs globally, being more common in developed nations and less so in developing ones. It was traditionally viewed as a Western issue, but the rise of IBD in developing countries, attributed to urbanization and modernization, is altering this perspective [3].

CD impacts the mouth and the anus. On the other hand, UC specifically affects the rectum and the intestine [4]. In CD, the inflammation-related sores are transmural, which means they may affect all layers of the intestinal wall and are not continuous, with healthy sections of the intestine interspersed with the affected ones. In UC, the inflammation-related sores are continuous, typically affecting the rectum and the colon’s proximal part. Contrary to CD, the muscularis propria is not impacted in UC [5]. Both inherent and adaptive immune system mechanisms could play a role in the pathology of IBD. Immune cells discharge cytokines, enzymes that degrade proteins, and detrimental molecules referred to as free radicals, which collectively contribute to inflammation and the formation of ulcers [6].

IBDs can extend beyond the gastrointestinal (GI) tract, potentially affecting various organs in the body. These manifestations outside the GI tract are referred to as extraintestinal manifestations (EIMs) of IBD. In both CD and UC, the musculoskeletal system (such as peripheral and axial arthritis, enthesitis), skin conditions (including pyoderma gangrenosum, erythema nodosum, Sweet syndrome, and aphthous stomatitis), the hepatobiliary tract (specifically primary sclerosing cholangitis), and the eyes (episcleritis, anterior uveitis, and iritis) are among the most commonly affected areas [7].

While the precise origin of IBD remains unclear, there has been considerable progress in comprehending the development of this condition. Studies suggest that the onset of IBD is associated with elements such as the individual’s genetic susceptibility, the makeup of the gut microbiota, various environmental factors, and irregularities in the immune system [6].

The microbiota within the human gut forms the majority of the overall human microbiota. This microbiota is highly variable, and its variation is influenced by factors such as the environment, genetic makeup, and immune reactions. The term "microbiome" encompasses the microorganisms, their genetic material, and the environment they inhabit. Dysbiosis of the gut bacterial microbiota has been observed in individuals with IBD [3]. Dysbiosis is characterized by alterations in the gut microbiota, encompassing an increase in pro-inflammatory organisms and a decrease in anti-inflammatory organisms. Continuous dysbiosis can push different bodily equilibriums to the edge of a breakdown, eventually leading to local and systemic inflammatory reactions. Comprehensive studies have shown that inflammatory conditions, such as IBD, are instigated by microbiota dysbiosis [8]. However, it remains uncertain whether this bacterial imbalance is a trigger or an outcome of IBD, and the exact mechanisms by which it impacts IBD pathogenesis remain unclear [3]. A multitude of research has suggested that gut microbes play a crucial role in triggering and maintaining inflammation in the intestinal tissues of individuals suffering from IBD. The gut microbiota engages with the human immune system, prompting the maturation and function of immune cells. This is accomplished through three primary methods: provoking the secretion of mucin by intestinal goblet cells to preserve the structural integrity of the mucus layer and serve as a barrier; triggering the growth of intestinal mucosa-associated lymphoid tissue; and fostering the differentiation and maturation of immune cells, mainly through microbiota-guided adjustments that stimulate the development of isolated lymphoid follicles (ILFs) for innate defense and the activation of naive T and B cells [8].

Fecal and blood-based biomarkers are valuable tools for both diagnosing and managing IBD. Fecal indicators such as calprotectin and lactoferrin have been extensively researched for their effectiveness in identifying IBD, evaluating disease activity, and forecasting potential relapses. Antibodies targeting* Saccharomyces cerevisiae* and perinuclear antineutrophil cytoplasmic proteins are employed in diagnosing IBD, distinguishing between CD and UC, and anticipating the risk of complications associated with CD [9]. Significant alterations have been noticed in the dominant bacterial groups of IBD patients, specifically *Firmicutes* and *Bacteroidetes*, which comprise over 98% of the gut microbiota. The makeup of the gut microbiota emerges as a vital factor in the progression of IBD, indicating its potential use as a diagnostic and prognostic marker [10]. This raises certain important issues. Distinct microbial profiles are indeed associated with CD and UC, and they can serve as reliable predictors. The onset of IBD is primarily associated with a dysbiosis of the overall microbiota and the overgrowth of specific pathogens.

This review emphasizes the critical role of the gut microbiome in predicting the onset and prognosis of IBD. It highlights the move towards individualized medicine and the need for more research to comprehend the intricate interplay between the gut microbiota, immune system, and genetics in IBD. The review also highlights the promise of new treatments and the significance of a comprehensive strategy in handling this multifaceted and demanding ailment.

## Review

Methods

To maintain transparency, comprehensiveness, and methodological rigor in our article, we closely followed the Preferred Reporting Items for Systematic Reviews and Meta-Analyses (PRISMA) guidelines. Our systematic literature search was greatly aided by adhering to the PRISMA methodology. We took great care in combining and refining search terms to ensure the inclusion of all relevant studies. Only studies directly related to our research question were considered, guided by clearly defined criteria for inclusion and exclusion. The screening process followed the PRISMA protocol, and the studies meeting the eligibility criteria were selected after initial reviews of titles, abstracts, and full-text assessments.

For the database search protocol, we have meticulously designed a method to ensure a thorough exploration of relevant studies. We utilized four distinct databases (PubMed, Google Scholar, MedLine, and Science Direct) to identify relevant literature. Our search strategy involved using Boolean operators and Medical Subject Headings (MeSH) keywords to refine and focus the search results, as demonstrated in Table 1. By strategically employing "AND" and "OR" operators, we were able to combine search terms to cover a broad range of materials while still pinpointing relevance. MeSH keywords were used where appropriate to improve the accuracy of our search. The search strategy was tailored to match the syntax and structure of each database, ensuring the optimal retrieval of relevant studies.

**Table 1:** Utilization of different search strings across different databases

Database	Search String
PubMed	“Gut microbiota” OR “Gut flora” OR “Gut microbes” OR ["Gastrointestinal Microbiome/immunology"[Majr] OR "Gastrointestinal Microbiome/physiology"[Majr] ] AND Biomarkers OR ["Biomarkers/analysis"[Majr] OR "Biomarkers/blood"[Majr] ] AND Inflammatory bowel disease onset and progression OR Crohn’s Disease OR Ulcerative colitis OR ["Inflammatory Bowel Diseases/diagnosis"[Majr] OR "Inflammatory Bowel Diseases/enzymology"[Majr] OR "Inflammatory Bowel Diseases/microbiology"[Majr] ]
Google Scholar	"Gut microbiota" OR "Biomarkers" OR "Inflammatory Bowel Disease"
MedLine	“Gut microbiome” AND “Inflammatory Bowel Disease”
Science Direct	Keywords: Gut microbiota; Inflammatory Bowel Disease; Biomarkers

The screening process initially involved evaluating studies based on their titles, followed by a subsequent screening based on their abstracts.

Inclusion Criteria

In terms of inclusion criteria, we incorporated studies involving human participants that were published within the last five years. This allowed us to extract data from the most recent research. Both observational studies (including cross-sectional, case-control, and cohort studies) and clinical trials were considered eligible for inclusion, as they provided diverse data sources for analysis. We encompassed published articles from peer-reviewed journals and unpublished gray literature to reduce potential publication bias. Additionally, we specifically focused on studies published in English.

Exclusion Criteria

Conversely, for exclusion criteria, we did not include animal studies or in vitro experiments, as our primary focus was on studies involving human populations. Studies published more than five years ago were also excluded, ensuring that we focused on the most up-to-date research. Conference abstracts, posters, and presentations lacking full-text articles were excluded due to the limited availability of comprehensive data. Furthermore, unpublished dissertations and theses were omitted to maintain consistency with the inclusion of peer-reviewed literature.

**Figure 1:**
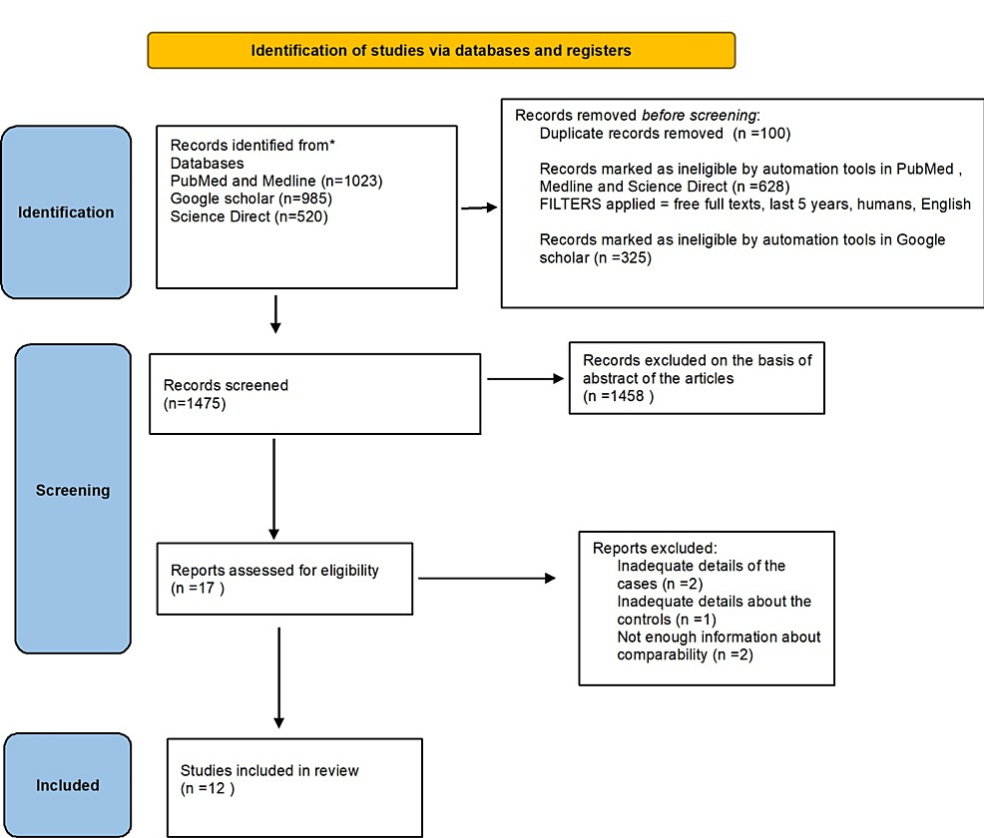
PRISMA flow diagram explaining the data extraction process PRISMA: Preferred Reporting Items for Systematic Reviews and Meta-Analyses

In order to guarantee the comprehensiveness and methodological precision of the study, we rigorously followed the PRISMA criteria, as depicted in Figure 1.

Result

Table 2 summarizes the studies in this review.

**Table 2:** A concise overview of the studies included in this systematic review

Author	Year of publication	Number of patients	Type of study	Subheading studied	Result/conclusion	P-value and association
Ocansey et al. [10]	2022	8,432	Cohort study	Pathogenesis	Mucolytic bacteria, particularly *Ruminococcus gnavus* and *Ruminococcus torques*, have been found to increase significantly in Crohn’s disease (CD) and ulcerative colitis (UC).	0.02, significant association
Guo et al. [11]	2022	734	Case-control study	Microbiota dysbiosis in IBD and potential clinical applications	The quantity of *Faecalibacterium prausnitzii* was diminished in individuals with active Crohn’s disease (CD) and ulcerative colitis (UC) compared to those in a state of remission. *Faecalibacterium prausnitzii* could potentially serve as a dependable indicator for evaluating the severity of the disease.	0.01, significant association
Haneishi et al. [12]	2023	535	Cohort study	Pathogenesis and clinical applications	Patients with inflammatory bowel disease (IBD) and mice modeled with colitis exhibited changes in their gut microbial compositions when compared to healthy individuals and mice. This implies that interventions such as probiotics and fecal microbiota transplantation (FMT) could potentially alleviate IBD symptoms by targeting the gut microbiota.	0.03, significant association
Serrano-Gómez et al. [13]	2021	1638	Cohort study	Microbiota dysbiosis in IBD	Crohn’s disease (CD) exhibits a higher level of dysbiosis than ulcerative colitis (UC), evident at both taxonomic and functional levels. The synthesis of short-chain fatty acids (SCFAs), including propionate, varies between CD and UC/healthy control (HC) subjects.	0.02, significant association
Khan et al. [14]	2019	746	Cohort study	Pathogenesis and clinical applications	Detailed research carried out on ulcerative colitis (UC) and Crohn’s disease (CD) patients has demonstrated a distinct decrease in *Firmicutes* (particularly groups of *Clostridium*) and a rise in *Proteobacteria*. An obvious role of the gut microbiota in host physiology has been demonstrated by the link between gut microbiota dysfunction and inflammatory bowel disease.	0.04, significant association
Caparrós et al. [15]	2021	174	Case-control study	Pathogenesis	The gut microbiota governs the equilibrium between Th17 and Treg cells. An altered reaction of CD4+ T cells to antigens derived from the microbiota could potentially trigger a proinflammatory response within the intestinal microenvironment contributing to IBD.	0.01, significant association
Foppa et al. [16]	2023	784	Case-Control study	Microbiota dysbiosis in IBD and pathogenesis	This article indicates that genetic variations, genetic predisposition, and the microbiota environment are linked to the pathogenesis of inflammatory bowel disease (IBD) through changes in the immune system and the composition of the microbiota.	0.03, significant association
Zheng et al. [17]	2022	2045	Case-control study	Pathogenesis and clinical applications	This research has revealed consistent changes in the composition of the gut microbiome that play a part in the development of inflammatory bowel disease (IBD). These findings have illuminated the potential significance of microbiome biomarkers in diagnosing IBD and forecasting its course.	0.06, no significant association
Axelrad et al. [18]	2020	9401	Cohort study	Microbiota dysbiosis in IBD and pathogenesis	Bacteria, specifically species of *Salmonella*, *Campylobacter*, and *Clostridioides difficile*, showed a steady positive correlation with the risk of developing inflammatory bowel disease (IBD). On the other hand, *Helicobacter pylori* and helminth infections were generally associated with a consistent decrease in the risk of IBD.	0.03, significant association
Wu et al. [19]	2023	564	Case-control study	Microbiota dysbiosis in IBD and clinical applications	The imbalance of gut microbiota has been associated with the cause of IBD; hence, addressing this imbalance appears to be a hopeful new treatment strategy.	0.07, no significant association
Al-Rashidi et al. [20]	2022	954	Cohort study	Microbial dysbiosis in IBD and clinical applications	Any disruption in the balance of gut microbes leads to a condition called dysbiosis. Consequently, the ability to resist the colonization of pathogens diminishes, leading to the favored growth of potentially harmful microbes, known as pathobionts, and an abnormal immune response.	0.03, significant association
Ma et al. [21]	2022	346	Cohort study	Microbiota dysbiosis in IBD	The gut microbiota in patients with early-stage Crohn’s disease is marked by a notable reduction in short-chain fatty acid (SCFA)-producing bacteria, including *Blautia*, *Clostridium IV*, *Coprococcus*, *Dorea*, *Fusicatenibacter*, and a significant increase in *Escherichia*/*Shigella* and *Proteus*, when compared to healthy individuals.	0.01, significant association

The studies reviewed encompassed a total of 25,402 patients. Of the eight studies analyzed, five were cohort studies, and three were case-control studies. The initial number of studies identified on PubMed and MedLine was 1023. A total of 520 articles were identified on Science Direct, while Google Scholar yielded 985 articles. After applying filters such as free full texts, systematic reviews, articles from the last five years, humans, and English on PubMed, 628 records were marked as ineligible by automation tools. On Google Scholar, applying the filter for articles since 2015 led to 325 records being marked as ineligible. In total, 100 duplicate records were removed.

A total of 1475 records were examined. After evaluating eligibility, 17 reports were taken into consideration. The review ultimately incorporated 12 studies, with five articles excluded due to insufficient details regarding cases, controls, and comparability.

The article proposes that the gut microbiota could serve as potential noninvasive biomarkers for assessing the activity of IBD. It emphasizes that understanding the relationship between the microbiome and disease activity could lead to the creation of a set of indicators derived from the microbiota. These indicators could be used to monitor the start and progression of IBD. The article also explores the possibility of changes in the gut’s microbial composition acting as early warning signs for the onset and progression of IBD.

Discussion

In recent years, the diagnosis and management of IBD have undergone significant changes. Studies that have observed the outcomes of IBD have shown a decrease in the number of surgeries performed, which is likely due to the development of new treatments. Approximately one-third of the patients with gastrointestinal symptoms do not show any evidence of mucosal inflammation. This highlights the need for more precise markers to aid in clinical diagnosis [11].

The interaction between the gut microbiota and autoimmune systems has been identified as a significant factor in the development of IBD. Studies have shown that the gut microbiota can initiate IBD events. The gut microbiota composition differs between healthy individuals and those with IBD [12]. Microbial biomarkers have the potential to aid in evaluating disease severity and predicting response to intervention. The gut microbiota may help clinicians define patient risk of postoperative relapse. Studies suggest that intestinal bacteria may be helpful in monitoring disease progression and predicting the development of IBD in patients [11].

Pathogenesis

The gut microbiota is responsible for maintaining homeostasis in the body by performing various functions such as fermenting complex undigested polysaccharide polymers, producing short-chain fatty acids (SCFAs), synthesizing certain vitamins, producing energy, maintaining the integrity of intestinal mucosa, and preventing pathogenic microbes. For instance, *Roseburia* and species from the* Lachnospiraceae *family are responsible for generating SCFAs, such as butyrate, acetate, and propionate, from fermentable dietary fiber. SCFAs play a crucial role in preserving the balance of the gut environment. Some of these SCFAs have the ability to reduce inflammation and enhance the robustness of the intestinal barrier. The species contributing to the production of SCFA varied significantly in CD and UC patients, with the contribution of species from the *Firmicutes* phylum in UC and species belonging to* Proteobacteria* in CD patients [13]. These fatty acids serve as a carbon source for intestinal colonocytes and aid in the differentiation of regulatory T cells (Tregs) [14,15]. Specific members of the symbiotic gut microbiota have been discovered to exert particular influences on the host’s immune system, which are vital for maintaining immune equilibrium. This comprehensive systematic review shows a significant and relevant association between gastrointestinal infections (gastroenteritis) and new-onset IBD. The review found biologically plausible mechanisms of action that support this association [15].

In patients with IBD, dysbiosis or imbalance in the gut microbiota leads to a disruption between beneficial and detrimental bacterial taxa. This, in turn, results in harm to the intestinal epithelial barrier, which comprises mechanical, chemical, immune, and microbial defenses. The gut microbiota can prompt T cells to diversify, mainly into Th17 and Treg cells, thus affecting the intestinal microenvironment. The inflammatory reaction driven by Th cells protects the host from detrimental pathogens. However, excessive activation of Th cells is associated with the initiation and progression of intestinal inflammation [16]. Similarly, a recent analysis of 132 patients with IBD conducted by Khan et al. demonstrated that the equilibrium between Th17 cells, known for their pro-inflammatory cytokines, and Treg cells, recognized for their anti-inflammatory cytokines, is vital for maintaining intestinal stability in the host. This balance is directly influenced by the standard content of the gut microbiota. Certain bacterial species from the *Clostridia *and* Bacteroides* genera have been found to induce anti-inflammatory and Treg cell responses [14].

An article by Zheng et al. has shown that certain microorganisms have a role in promoting or inhibiting inflammation, forecasting the reaction to treatment, and assessing the likelihood of relapse post-surgery in patients with IBD. According to these studies, specific microbial signatures can be used to diagnose IBD. These signatures are more likely to be present in higher levels in cases than in controls, making them a reliable diagnostic tool. According to a study by Lopez-Siles et al., *Faecalibacterium prausnitzii* is most effective in distinguishing between healthy subjects and CD patients [16]. *Faecalibacterium prausnitzii *in healthy individuals can suppress the NFκB pathway via the microbial anti-inflammatory molecule (MAM). Additionally, the production of interferon-gamma (IFN-gamma) and interleukin-12 (IL-12) by dendritic cells is also reduced in the presence of *F. prausnitzii*, which encourages the production of IL-10 under stable conditions, thus contributing to its anti-inflammatory effect [15].

Dysbiosis is a term utilized to depict any change in the structural arrangement of the gut microbiota that can disturb the microbial equilibrium. This disruption is related to various gut pathologies and inflammation of the intestines. When the balance of the gut microbiota is disturbed, it can lead to changes in the functions of the gut microflora. These changes can affect the production of fermentation products such as carbohydrates, vitamins, and SCFAs, as well as biochemical processes such as immune equilibrium imbalance [16]. Treg cells interact with the microbiota, and their proliferation is controlled by the byproducts of beneficial bacteria. The equilibrium between these Treg cells and adaptive Th17 cells (which primarily produce IL-17 and IL-22), along with the gut microbiota, plays a direct role in the onset or exacerbation of disease. An imbalance in the correlation between Th17/Treg and their production of pro-inflammatory cytokines can cause or propagate disease pathogenesis. This suggests a link between dysbiosis and disease etiology [17].

An article by Axelrad et al. cites a recent study carried out in Sweden that examined 480,721 patients and discovered that a diagnosis of any form of gastroenteritis was linked to higher chances of developing IBD. Several studies referenced by Axelrad et al. have shown that *Mycobacterium avium paratuberculosis* (MAP) may be involved in CD pathogenesis. A minor investigation of T-cell lines from intestinal samples of 11 CD patients, 13 UC patients, and 10 controls found that 71% of active CD patients had a strong Th1/Th17 response to MAP. This suggests that mycobacteria may play a role in CD, as MAP-reactive CD4 T-cell clones synthesized IL-17 and/or IFN-gamma [18].

Research conducted by Ocansey et al., referencing genetic studies involving a large group of 86,640 patients with IBD and controls, has identified 38 locations in the genome that influence IBD risk. Alterations in a number of these genes frequently affect particular host mechanisms associated with the microbial reaction in IBD, such as the bacterial infection response driven by NOD2 innate immunity [10]. NOD2, the initial gene identified as being susceptible to IBD, is a NOD-like receptor (NLR) that attaches to bacterial muramyl dipeptide. Paneth cells in the intestine can detect bacteria through intracellular NLRs and exercise their influence by producing antimicrobial peptides. A decrease in the production of these antimicrobial peptides could change the makeup of the microbiota, potentially leading to a heightened vulnerability to inflammation [15].

Specific bacterial clusters (*Bacteroidaceae, Roseburia, and Faecalibacterium prausnitzii*) were discovered to participate in NOD2 signaling, whereas one (*Firmicutes*) was linked with *CARD9*. Specific forms of the *CARD9* gene, while generally raising the risk of IBD, can surprisingly offer some patients protection. This phenomenon is linked to changes in the gut microbiome, particularly involving bacteria such as *Citrobacter rodentium, Firmicutes,* and *Clostridiaceae*. These findings suggest that gut bacteria not only contribute to the characteristic dysbiosis observed in IBD but also to host genetic mutations that promote and maintain the disease [10].

The article also cites a study that reported an increased frequency of virulence factors (gelatinase (gelE)) in *Enterococcus* strains isolated from patients with IBD compared to healthy controls. The researchers inferred that those strains of *Enterococcus*, which firmly attach to the intestinal lining, create biofilms, and have antioxidant protective systems, seem to exert the most substantial influence on the inflammation process. Although mucolytic bacteria exist in the gut of healthy individuals and serve as a crucial component of the bacterial group associated with the mucosa, their quantities are said to rise in IBD. This research observed an average increase of 1.9-fold in mucolytic bacteria in CD and 1.3-fold in UC. Specific bacteria such as *Ruminococcus gnavus* and *Ruminococcus torques* saw increases of more than four-fold and approximately 100-fold, respectively [10].

In summary, based on these findings, specific intestinal infections and their subsequent effects (for instance, metabolomic profiles) could conceivably initiate gut dysbiosis, serve as surrogate markers to identify patients with existing dysbiosis, aggravate existing dysbiosis, induce immunological scarring that alters subsequent immune reactions, or directly harm the intestinal barrier and trigger mucosal immune responses. All these factors contribute to the pathogenesis of preclinical and clinical IBD or IBD relapse in genetically predisposed individuals. Even though numerous clinical investigations that fulfilled the selection standards were prone to methodological constraints and prejudices that we have underscored, and absolute causation cannot be confirmed, these details still offer beneficial perspectives.

Microbial Dysbiosis in Inflammatory Bowel Disease

The standard microbiota in the gastrointestinal tract plays a crucial role in preserving a healthy intestinal setting and warding off infections caused by harmful bacteria. The standard microbiota is marked by its vast diversity, with a dominance of *Firmicutes* and *Bacteroidetes* and a minimal presence of *Enterobacteriaceae* [11]. Alterations in the composition of gut microbes are frequently seen in patients with IBD. There is a significant overlap in the gut microbial patterns of UC and CD. They definitely displayed some unique microbial markers. The microbial shifts in CD patients were typically more pronounced than those in UC patients [19]. In comparison to healthy individuals, the quantities of beneficial bacteria, such as *Faecalibacterium prausnitzii* and *Roseburia intestinalis, *are notably diminished in CD and/or UC. Conversely, the relative abundance and proliferation of bacteria such as *Gamma proteobacteria and Enterobacteriaceae (for instance, Escherichia coli)* are elevated [16,19,20]. This was supported by Guo et al., who evaluated the presence of *F. prausnitzii* and *E. coli* in a group consisting of 28 healthy individuals, 45 patients with CD, 28 patients with UC, and 10 patients with IBS. The results affirmed that *F. prausnitzii *is a specific marker for IBD. The prevalence of* F. prausnitzii* in patients with IBD was significantly less than in IBS patients and healthy individuals (P < 0.001) [11].

**Figure 2:**
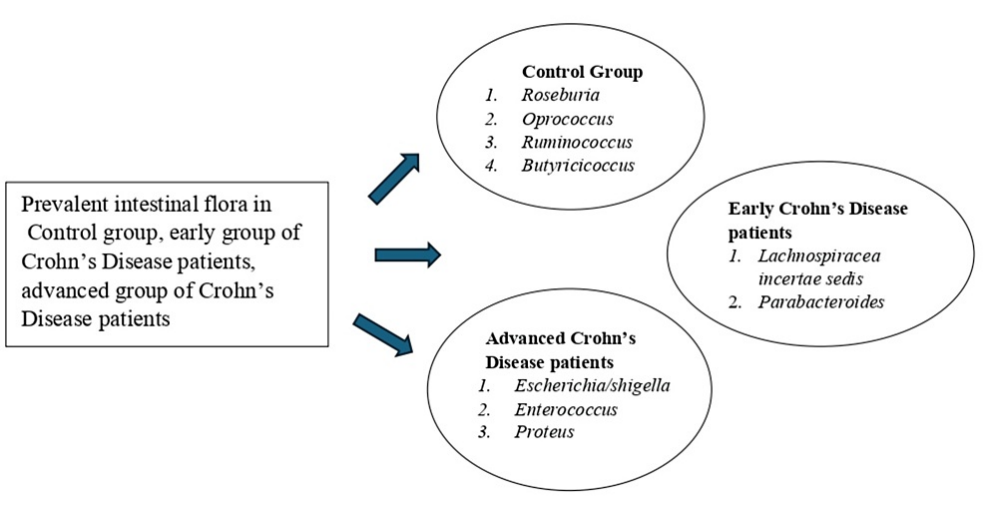
The intestinal microbiome of the control group, early CD patients, and advanced CD patients CD: Crohn's disease

When *F. prausnitzii* is considered in conjunction with *E. coli*, it can even differentiate colonic CD from extensive colitis, suggesting that a combination of multiple bacteria may serve as more appropriate biomarkers for IBD than individual bacteria. A meta-analysis using a random-effects model, which included 231 CD patients and 392 UC patients, demonstrated that* F. prausnitzii *was lower in IBD patients compared to those in remission. According to Ma et al., distinct compositions of microbiota were observed in samples from CD patients (including those in early and advanced stages) compared to samples from healthy individuals. In patients with CD, there is an observed increase in certain species, such as* E. coli*, *R. gnavus*, and *C. clostridioforme*. On the other hand, there is a noticeable decrease in *F. prausnitzii*. These changes are in comparison to both UC patients and HC [13]. Notably,* Lachnospiracea incertae sedis* and *Parabacteroides* were more prevalent in patients in the early stages of CD, while *Escherichia*/*Shigella*,* Enterococcus*, and *Proteus* were more prevalent in patients in the advanced stages of CD. Moreover, the results from the Kruskal-Wallis rank sum test revealed that *Clostridium IV*, *Coprococcus*, and *Fusicatenibacter* continued to decline in both early and advanced CD patients. In contrast, *Escherichia*/*Shigella *and *Proteus* continued to increase significantly compared to HC (P < 0.05) [21]. Figure 2 illustrates variations in the gut microbiome in the control group, early CD patients, and advanced CD patients.

The traits of the gut microbiota were further scrutinized in various disease activity categories among patients with IBD. There was a significant increase in *Proteobacteria* and *Enterococcaceae* and a decrease in *Ruminococcaceae *and *Clostridiales *in patients with moderate to severe CD. Importantly, most of these microbiota differences related to disease activity were associated with the *Firmicutes*, *Bacteroidetes*, and *Proteobacteria* phyla [2].

In summary, this systematic review found that the diversity of the gut microbiota in IBD patients was lower compared to healthy individuals, with a nonsignificant trend indicating a more pronounced reduction in UC patients than in CD patients [2]. This article also demonstrated that the presence of* F. prausnitzii* was lower in patients with active CD and UC compared to those in remission. This further suggests that *F. prausnitzii* could be a reliable indicator for evaluating disease activity [11]. In contrast, there was a significant increase in *Escherichia*/*Shigella *and *Proteus* compared to healthy individuals. This suggests that *Escherichia/Shigella* might play a crucial role in the progression of CD. The findings of this article bolster the notion that the gut microbiota harbors potential biomarkers for the noninvasive evaluation of IBD onset and progression. These insights could potentially contribute to the development of guidelines for IBD treatment. However, this hypothesis requires further investigation.

Potential Clinical Applications

Recent advances in microbiome research have highlighted the potential of gut microbiota as a tool for detecting the onset and progression of IBD. Alterations in the structure and role of the gut microbiota, such as a reduction in favorable bacteria and a rise in potentially detrimental ones, can act as markers of disease progression. By monitoring changes in the gut microbiota, clinicians may be able to detect IBD at an early stage, track its progression, and even assess the effectiveness of treatments [17].

Studies have suggested that a disruption in the gut microbiome could contribute to the activity of IBD disease. An extensive analysis showed that the quantities of *Clostridium leptum*, *F. prausnitzii*, and *Bifidobacterium* were diminished in patients with CD and UC who had active disease status in comparison to those in remission. However, the presence of *Clostridium coccoides *bacteria was less in UC patients with active symptoms, while CD patients did not show this change. Also, *Streptococcus* bacteria were more abundant in samples from CD patients who experienced disease recurrence after surgery. Sokol et al. reported that a decrease in alpha diversity and an increase in the *Proteobacteria *phylum were associated with endoscopic recurrence of CD, along with a corresponding decrease in members from the *Lachnospiraceae* and *Ruminococcaceae* families within the *Firmicutes* phylum [17].

It has also been demonstrated that the gut microbiota and specific taxonomic features of bacteria can influence the response and outcome of drug treatments. In a prospective study involving serial fecal sampling from CD patients who were starting anti-integrin inhibitors, Ananthakrishnan and colleagues discovered that patients who achieved remission had greater α-diversity and a higher abundance of *Roseburia inulinivorans* and *Burkholderiales* species at the outset. Zheng et al. reference a study by Zhou et al. that demonstrated that microbial taxonomy, primarily *Clostridiales*, has a high predictive ability in determining the response to infliximab treatment. Combining calprotectin and CDAI with the test dramatically increased its accuracy from 86.5% to 93.8%. Adding calprotectin and CDAI boosted the test's accuracy by a noteworthy 7.3 percentage points, bringing it to 93.8%. In a comprehensive review of 19 studies, it was observed that individuals with IBD who responded to exclusive enteral nutrition, infliximab, ustekinumab, or vedolizumab had increased baseline gut bacteria α-diversity. Additionally, we observed that people who improved with aminosalicylates, anti-TNF drugs, and ustekinumab medication had higher levels of *F. prausnitzii* bacteria. These findings underscore the significance of the gut microbiome composition in determining the treatment response in IBD [17].

Research conducted by Guo et al. suggests that alterations in the gut microbiota can accurately identify patients with CD who are responsive to TNF therapy. This (identification of distinct gut microbiota profiles in TNF-responsive CD patients) could facilitate the optimization of clinical management, mitigate the incidence of morbidity, and alleviate the symptomatology for this patient population. In a study by Sanchis-Artero and colleagues, 27 CD patients initiating anti-TNF treatment were divided into responders and nonresponders. The researchers evaluated the ratios of *F. prausnitzii*/*E. coli* and* F. prausnitzii*/*C. coccoides* before and after six months of treatment. The findings indicate that the *F. prausnitzii*/*E. coli* ratio could serve as a reliable early biomarker for identifying anti-TNF responsiveness in patients with CD [11].

Under normal physiological circumstances, the gut microbiota functions as a homeostatic organ. It plays a role in the fermentation of complex, undigested polysaccharide polymers, the production of SCFAs, the synthesis of certain vitamins, energy generation, the maintenance of intestinal mucosa integrity, and the prevention of pathogenic microbes [14].

Administration of VSL capsules in preclinical animal models of IBD has been shown to ameliorate disease symptoms via modulation of the intestinal milieu, characterized by enhanced microbial diversity and normalized abundance of specific commensals, such as *Bifidobacterium *and *Turicibacter*. Furthermore, the use of VSL capsules has been shown to improve inflammatory responses in the intestine. This suggests that the restructuring of the gut microbiota composition by VSL may have inhibited intestinal inflammation, thereby alleviating IBD pathologies. Moreover, numerous clinical studies have indicated that VSL is beneficial in improving IBD. While the safety of VSL is acknowledged, the concerns related to its use in clinical applications are yet to be fully understood. Therefore, confirming the effectiveness of VSL could pave the way for its widespread use in the treatment of IBD [12].

The gut microbiota exhibits metabolic potential through the production of a repertoire of bioactive metabolites. These molecules are absorbed into the enterohepatic circulation and, subsequently, the systemic circulation, which profoundly affects host energy homeostasis, inflammatory responses, endocrine regulation, and metabolic programming. In order to elucidate the functional significance and interindividual variability of microbiota-derived metabolites in IBD, a multi-omic approach was employed. This involved metagenomic profiling of gut microbial communities alongside targeted and untargeted metabolomic analyses of fecal, urinary, and serum samples obtained from both healthy controls and IBD patients. A metabolomics analysis of fecal samples from 155 subjects with CD in the USA and 65 healthy controls in the Netherlands revealed significant increases in eight fecal metabolites. Among these, sphingolipids, carboximidic acids, and bile acids exhibited the most prominent alterations, suggesting potential biomarkers for disease status [17].

Haneishi et al. stated that fecal microbiota transplantation (FMT) not only ameliorated the pathogenesis of colitis in mice with gut dysbiosis but also notably elevated the levels of intestinal bacterial metabolite SCFAs [12]. SCFAs, which act as an essential energy supply for intestinal epithelial cells (IECs) and are recognized to enhance the performance of the gut barrier, were generally observed at reduced levels in the fecal microbiota of patients with active IBD. This aligns well with the reduction of SCFAs-producing bacteria, particularly those belonging to the phylum *Firmicutes*. A drop in *F. prausnitzii*, a specific gut bacterium important for butyrate production, is a hallmark of active IBD. FMT has the potential to counter these changes and increase the *Clostridium *cluster, including *F. prausnitzii* [19]. SCFAs fight inflammation and improve immunity, leading to potential benefits for IBD, as shown by Zhang et al.'s study on FMT [12]. The conceptual foundation for employing FMT is rooted in the notion that transplanting fecal matter from a healthy donor into the recipient’s gut can reestablish a more balanced and varied microbiome, potentially easing symptoms and facilitating recuperation [16]. A double-blinded study tested whether FMT could improve UC symptoms. Patients with active UC received either FMT or a placebo. The study found changes in the gut microbiota of the FMT group. Therefore, the changes in the gut microbial composition induced by FMT may at least partially alleviate the signs and symptoms of IBD [12].

In summary, this article demonstrates a consistency in the imbalance of the gut microbiome in IBD patients across various cohorts and ethnic groups. These insights could potentially assist in formulating principles for IBD treatment, such as FMT, that can alter the gut microbiome and help in easing the signs and symptoms of IBD.

Limitations

Several constraints of this study should be recognized. Initially, the incorporation of numerous case-control studies with comparatively small sample sizes could influence the applicability of the results. Moreover, this study concentrated solely on articles published in the English language, potentially overlooking pertinent research conducted in other languages. Finally, the choice of papers published only within the recent five years may have confined the historical perspective of the analysis. The limited availability of free full-text articles might lead to the potential exclusion of valuable research papers.

While our analysis identified promising results within these clinical studies, the existence of methodological limitations and potential biases prevents us from definitively claiming a causal relationship between the intervention and the observed outcomes. However, these data still provide valuable insights into our understanding of the potential clinical applications of the gut microbiome for predicting IBD onset and prognosis.

## Conclusions

This review highlights the crucial role of gut microbiota dysbiosis in IBD pathogenesis and progression. IBD patients exhibit less gut microbiota diversity than healthy individuals, with a slightly greater decrease in UC than CD patients. Changes in the gut microbiota, such as decreased beneficial and increased detrimental bacteria, can serve as disease progression markers. Monitoring these changes can help detect IBD early, track its progression, and assess treatment effectiveness. The study supports the potential of gut microbiota as noninvasive biomarkers for IBD evaluation, diagnosis, and prognosis. The identification of a disease activity-associated microbiome paves the way for microbiota-based indicators for IBD progression assessment and treatment guidelines, including FMT. However, further research is needed to confirm causality and the underlying mechanisms.
